# The relationship between epilepsy and cognitive function in benign childhood epilepsy with centrotemporal spikes

**DOI:** 10.1002/brb3.1854

**Published:** 2020-09-22

**Authors:** Yihan Li, Yulei Sun, Tingting Zhang, Qi Shi, Jintao Sun, Jing Xiang, Qiqi Chen, Zheng Hu, Xiaoshan Wang

**Affiliations:** ^1^ Department of Neurology Nanjing Brain Hospital Nanjing Medical University Nanjing China; ^2^ MEG Center Division of Neurology Cincinnati Children’s Hospital Medical Center Cincinnati OH USA; ^3^ MEG Center Nanjing Brain Hospital Nanjing China; ^4^ Department of Neurology Nanjing Children’s Hospital Nanjing China

**Keywords:** benign childhood epilepsy with centrotemporal spikes, functional connectivity, graph theory, magnetoencephalography, multi‐frequency, neural network

## Abstract

**Introduction:**

This study was aimed to explore the relationship between neural network changes in newly diagnosed children with Benign Childhood Epilepsy with Centrotemporal Spikes (BECTS) and cognitive impairment.

**Methods:**

Children's cognition was evaluated using the Wechsler Intelligence Scale for Children‐Fourth Edition (WISC‐IV). Magnetoencephalographic (MEG) data of 18 healthy children and 22 BECTS patients were recorded in order to construct a functional connectivity (FC) network, which was quantified by graph theory (GT).

**Results:**

The mean age of the control group was 7.94 ± 1.89 years, and the mean age of BECTS patients was 8.14 ± 1.73 years. Our results show that the WISC‐IV index scores in the BECTS group were significantly lower than those in the control group. Besides, the FC network pattern of BECTS patients changed significantly in the 12–30, 30–80, and 250–500 Hz frequency band. The local functional connections between posterior cingulate cortex (PCC) and frontal lobe varied significantly in 12–30, 80–250, and 250–500 Hz. Our GT analysis shows that the connection strength of BECTS patients increases significantly in the 12–30 Hz frequency band, the path length decreases significantly in the 12–30 Hz and 30–80 Hz frequency bands, with the clustering coefficient decreasing significantly in the 12–30 Hz, 30–80 Hz, and 250–500 Hz frequency bands. Correlation analysis showed that the full‐scale IQ (FSIQ) was positively correlated with the 12–30 Hz clustering coefficient, verbal comprehension index (VCI) was positively correlated with the 250–500 Hz clustering coefficient, perceptual reasoning index (PRI) was positively correlated with the 12–30 Hz clustering coefficient, and perceptual reasoning index (PSI) was negatively correlated with the 12–30 Hz path length.

**Conclusion:**

There is a trend of cognitive impairment in patients with early BECTS. This trend of cognitive impairment in early BECTS children may be related to the changes in the FC network pattern.

## Graphical abstract

BECTS patients have a certain degree of cognitive dysfunction in the early stage of the disease. The functional connections of patients with early BECTS showed unbalanced brain topology, and the brain network pattern was tending to be randomized. This trend of cognitive decline in early BECTS children may be related to the change of FC network patterns.

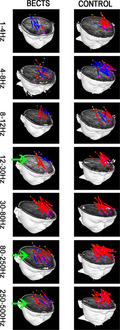

## 1 INTRODUCTION

Self‐Limited Childhood Epilepsy with Centrotemporal Spikes, previously known as Benign Childhood Epilepsy with Centrotemporal Spikes (BECTS), is one of the most common epilepsy syndromes in childhood, accounting for approximately 15% to 24% of children with epilepsy (Wirrell, 1998). The age of onset for BECTS is 2 to 14 years old, with 5 to 10 years of age being the most common (Wirrell, 1998). Benign Childhood Epilepsy with Centrotemporal Spikes is a type of partial epilepsy that is age‐related and characterized by seizures that gradually disappear with age (Wirrell, 1998). Its etiology is still unclear; however, it appears that it may have a genetic predisposition, in addition to being closely related to sleep (Samaitienė et al., 2013). Moreover, studies using electroencephalogram (EEG) analysis have shown localized discharges in the central and temporal regions of the brain (Parisi et al., 2012). Notably, traditional beliefs are that children with BECTS have no neurological abnormalities or cognitive impairment, and that seizures stop by themselves during puberty. However, recent studies have shown that BECTS children have some degree of cognitive impairment, behavioral and social impairment, and psychotic comorbidities (Eom et al., 2014; Neri et al., 2012; Völkl‐Kernstock et al., 2009; Yan et al., 2017), with speech impairment and executive dysfunction being the most prominent (Neri et al., 2012; Parisi et al., 2012).

It has been previously shown that BECTS patients have significantly lower cognitive function than normal children (Wickens et al., 2017). For instance, some scholars found that regardless of the type of epilepsy syndrome, school‐age children with new‐onset epilepsy scored worse in the cognitive domain test, when compared to the control group (Hermann et al., 2006), although there are diverse opinions on this matter. In most studies, children with BECTS have cognitive impairment and perform worse in certain cognitive areas; for instance, Jurkeviciene et al. assessed the language function of 61 children with BECTS VS. 35 children without BECTS as controls. Their results showed that children with BECTS performed worse than children from the control group, particularly in speaking fluency, naming speed, and instruction comprehension tasks (Eom et al., 2014). It was also shown that children with an earlier age onset, presented lower language scores. Given the high unmet need for children with BECTS, cognitive impairment due to BECTS is an important area of research and for the development of novel treatment options.

BECTS is characterized by few clinical seizures, such as sudden loss of consciousness with convulsion of face or limb, although the discharge of repeated spikes is often frequent (Clemens & Majoros, 1987; Drury & Beydoun, 1991; Wirrell, 1998). Currently, most authors believe that the clinical discharge, especially the frequent discharge of repeated spikes during sleep, is the main cause of cognitive impairment in BECTS, not the seizure itself (Samaitienė et al., 2013; Vannest et al., 2015). In recent years, understanding the molecular mechanisms underlying the characteristic epilepsy discharge of BECTS and its associated cognitive impairment has attracted the attention of many researchers. They believe that discharges of repeated spikes can cause subtle changes in brain tissue structure and its functional connections (Ciumas et al., 2014; Vannest et al., 2013), thus resulting in changes in the brain network (Kim et al., 2014; Luo et al., 2016). These changes may lead to disorders in otherwise normal neural regulatory networks (Lillywhite et al., 2009; Wickens et al., 2017) and may be related to the cognitive impairment observed in BECTS patients.

During the resting state, the brain is also active in an organized way. This mode is called the brain's default mode network (DMN), which mainly includes the posterior cingulate cortex (PCC), the dorsal medial prefrontal cortex (middle frontal gyrus, superior frontal gyrus), dorsal anterior cingulate gyrus, and bilateral angular gyrus, among others (Parisi et al., 2012). Repeated spikes may damage the brain's internal neural connections, leading to changes in DMN. Many previous studies have shown that there is a change in the DMN in children with BECTS which may cause the cognition decline, and PCC and frontal lobe are the key brain regions that constitute DMN (Chen et al., 2019; Chen et al., 2018; Maldjian et al., 2014).

Magnetoencephalography (MEG) is a comparatively new clinical imaging technique that is much appropriate for localization of neuromagnetic signals (Hämäläinen, 1992). MEG is a noninvasive detection tool, and it is considered superior to scalp EEGs, as magnetic signals can pass through skull, skin, and other tissues without significant distortion (Barkley & Baumgartner, 2003; Wheless et al., 2004). Magnetoencephalography and graph theory (GT) have been used in earlier studies to reveal the topology of the brain's internal functional networks (Maldjian et al., 2014). In addition, the characteristics of the changes occurring in resting states have been reported to act as indicators of the progression of various diseases (Olde Dubbelink et al., 2014; Ye et al., 2014). Through multi‐frequency MEG and GT analysis, the neural network's topological characteristics and specificity in different frequency bands can be systematically demonstrated.

Based on the above research data, we hypothesized that children with early BECTS may perform lower cognition than healthy children and that the associated network connections may change. Our study used the fourth edition of the Wechsler Intelligence Test to evaluate the cognition of BECTS patients. To this end, we used MEG to observe global FC neural changes in BECTS patients' brains, who were not taking antiepileptic drugs (AEDs). Considering the core position of frontal lobe and PCC in DMN, we intercepted local brain functional connections emitted from the PCC to the frontal lobe of the whole brain FC network and analyzed the partial functional connections of the brain FC network. Besides, we explored the correlation between cognitive function changes and FC network changes.

## 2 EXPERIMENTAL PROCEDURES

### 2.1 Subjects

We chose 24 BECTS patients from age 6 to 13 in two hospitals in Nanjing, China. 4 of them came from the Neurology Department of the Nanjing Brain Hospital, the other 20 came from Nanjing Children's Hospital. None of the patients were taking AEDs. For comparison, we recruited a total of 26 healthy children for the control group. The patients’ educational conditions, family background, as well as their parents' educational background and income were taken into consideration, from which 18 healthy children were selected with age, gender, parents' educational background, and family socioeconomic matching. All patients met the criteria for seizures according to ILAE 2017 classification (Fisher et al., 2017). A total of 24 BECTS patients were screened, but only 22 met the inclusion criteria and were included in this research. A total of 18 healthy controls were included in the analysis, all of which were screened and met the inclusion criteria. Clinical patient details are shown in Table 1. The research protocol was ratified by the Medical Ethics Committee of Nanjing Medical University, Nanjing Brain Hospital, and Nanjing Children's Hospital. All the children patients and their parents were informed of the research purpose and procedure and they all agreed to take part in the research. No patient subject had seizures 72 hours before the scan and 24 hours after the scan.

**TABLE 1:** Clinical patient's data

Patients	Sex	Age (years)	Course of epilepsy (months)	Number of seizures	Location of epileptic spikes (left, right, both sides)	Onset to scan (days since last seizure)
1	M	6	0.50	1	L	5
2	M	10	0.90	2	L	5
3	M	9	1.60	2	Both	3
4	F	8	0.90	1	R	3
5	M	9	0.20	1	R	3
6	M	11	0.70	2	L	3
7	F	12	1.50	2	L	3
8	F	6	0.90	3	L	3
9	F	10	2.10	2	R	5
10	M	10	0.80	1	Both	4
11	F	8	3.00	3	L	3
12	F	7	2.20	2	L	3
13	M	8	1.20	2	R	5
14	F	8	1.50	3	R	5
15	M	7	1.60	3	Both	3
16	F	6	0.70	2	Both	4
17	M	6	0.50	2	Both	3
18	M	8	0.70	2	L	3
19	M	6	0.20	1	R	4
20	F	7	0.20	1	L	3
21	F	9	1.90	3	R	4
22	F	8	2.10	4	R	3

Note

*F* = female; *M* = male.; L = left; R = right.

Here are the criteria for selecting a patient subject: (a) In line with the International League Against Epilepsy (ILAE) 2017 classification of epilepsy syndrome, children diagnosed with BECTS; (b) Routine clinical EEG examinations indicating high amplitudes originating from the central temporal region spike or the spike‐and‐slow wave complex with normal background waves; (c) Currently not using antiepileptic drugs; (d) Patients aged from 6 to 13 years, normal height and weight, receiving formal education, without other types of epilepsy, neurological diseases, or other major diseases, and with normal MRI scan results; (e) Parents of average intelligence, with a high school education or above; and (f) Parents / legal guardians and subjects willing to sign the informed consent as required.

Here are the exclusion criteria: (a) Metal implants like vagus nerve stimulation (VNS) devices producing significant nuclear magnetic noise on MEG data; (b) History of significant neurological or mental illness (e.g., brain trauma, schizophrenia); (c) A history of extensive developmental disorders; (d) Clinically significant systemic organ disease; (e) Patients who cannot follow and complete the study; and (f) Participants in drugs or device studies within 30 days of screening.

### 2.2 MEG recordings

A full‐head CTF275 channel MEG system (VSM MedTech Systems, Inc., Coquilam, BC, Canada) was used to record MEG signals in a magnetically shielded room, MEG Center of the Nanjing Brain Hospital. The solenoid was connected to the subject's nasal and left and right ear acupoints. These three coils were then activated to measure the subject's head position in relation to the MEG sensor. MEG data were then recorded for 120 s (s) at a sampling rate of 6,000 Hertz (Hz). All subjects were asked not to fall into sleep. During the recording time, subjects were asked to keep still and to close their eyes gently, not to think about anything. For each subject, at least 6 continuous data files with a duration of 2 min (min) were collected. The head position was measured at the beginning and at the end of each scan. We deleted the head movement in excess of 5 mm during recording and did re‐recording. In order to prevent having the effect of seizures on the MEG data results, we collected the data at least 3 days after the last seizure.

### 2.3 MRI scan

All subjects underwent an MRI scan with a 3.0 T scanner (Siemens, Munich, Germany), under the following conditions: a phase of the field of view of 100%, a scanning field of view of 240 µm, a repetition time (TR) of 6,600 µm, a layer thickness of 1.00 µm, and echo time (TE) of 93 µm. MRI markers were placed at the positions of the three coils used in the MEG recording to coregister MRI and MEG data.

### 2.4 Data preprocessing

All MEG recordings without long‐term artifacts were reserved. The MEG waveform signal was preprocessed through the following order: (a) Based on previous studies (Xiang, et al., 2015), MEG data with obvious noise and artifacts (amplitude > 6pT) are excluded; (b) Using filtered MEG data of the patient group without noise or artifacts in the 1–70 Hz band to clearly see the high amplitude spikes characteristic of BECTS; and (c) Considering the impact of spikes on network connection, in order to ensure the homogeneity and conduct a better comparison study with healthy children, 60 s continuous fragment of data without spikes were selected for the following data analysis. Correspondingly, a 60 s length fragment was selected as the control in the qualified MEG data of healthy children. The selected MEG data were then analyzed in 7 predefined frequency bands as follows: δ (1–4 Hz), θ (4–8 Hz), α (8–12 Hz), β (12–30 Hz), γ (30–80 Hz), ripple (80–250 Hz), and fast ripple (250–500 Hz). To avoid environmental alternating current power interference around the 50 Hz band, the corresponding filtering was performed before analyzing the data. All calculations on the MEG data were performed using a software‐MEG processor (https://sites.google.com/site/braincloudx/).

### 2.5 The Wechsler intelligence scale for children—fourth edition

Based on a previous study (Yang et al., 2013), we used the Chinese version of the Wechsler Intelligence Scale for Children 4th edition (WISC‐IV) in order to assess the intelligence level of the subjects (Wechsler, 2007). WISC‐IV is a separately managed normative reference tool designed to measure intelligence (Dumont & Willis, 2008). It contains 10 core subtests and 4 additional subtests, aggregated into four index scores and one full‐scaled IQ (FSIQ), ranging from lowest (40 points) to highest (160 points). The average standard score of the index is 100 points, with standard deviation is 15 points. The verbal comprehension index (VCI) includes vocabulary, similarity, and comprehension score tests (additional subtest: common sense test), the perceptual reasoning index (PRI) includes block design, picture concept, and matrix reasoning score tests (additional subtest: mapping test), the working memory index (WMI) includes numeric breadth and alphanumeric ranking testing (additional subtest: arithmetic test), and the processing speed index (PSI) includes coding and symbol search score tests (additional subtest: cancellation test). Importantly, all tests are performed by clinical psychologists. The FSIQ, four indicators, and subtests have shown good reliability (Baron, 2005).

### 2.6 Functional connectivity analysis

Based on previous reports (Xiang et al., 2013; Xiang, et al., 2015), we used wavelet‐based beamformer technology to project the collected MEG signal from the sensor level to the source level (Xiang et al., 2009, 2010). Functional connection was analyzed at the source level. First, the whole brain magnetic source was reconstructed using beamforming and other magnetic source imaging methods. The specific mathematical algorithm and effectiveness are described in detail by Xiang and colleagues (Xiang et al., 2013, 2014; Xiang, et al., 2015). This method has also been used in several related research articles (Miao et al., 2019; Tang et al., 2016; Wang et al., 2010; Wu et al., 2017). In order to analyze FC at the source level, the algorithm described above is used to calculate the virtual sensor waveform for each source. By analyzing the signal correlation of each pair of virtual sensors within the 60 s time window, the source neural network was estimated. Specifically, by calculating the correlation factor (or correlation coefficient), the relationship between the virtual sensor signals from the dual‐source pair is statistically analyzed. The correlation factors are defined as follows:

(1) $$R_{\left(X_{a},X_{b}\right)}=\frac{C\left(Xa,X_{b}\right)}{SX_{a}SX_{b}}$$

where $R_{\left(X_{a},X_{b}\right)}$ represents the correlation of the source pair at two positions ("a" and "b"). $X_{a}$ and $X_{b}$ represent signals from two sources, which are computationally connected in pairs. $C\left(Xa,X_{b}\right)$ represents the average of the two source signals. $SX_{a}$ and $SX_{b}$ represent the standard deviations of the two source signals. Notably, to avoid possible biases, we used a source‐level analysis to calculate all possible connections for each dual‐source pair. The FC distribution of each possible voxel‐based virtual sensor was collectively coregistered in the MRI of every single participant (Xiang, et al., 2015). The neural network based on magnetic source imaging allowed us to performed visual analysis on the axial, coronal, and sagittal planes. Thresholds were used as checkpoints to ensure the quality of the data. Finally, in order to determine the threshold of the connection, the *t*‐values of all source pairs were calculated:

(2) $$T_{P}=R\sqrt{\frac{K‐2}{1‐R^{2}}}$$

In equation (2), $T_{P}$ is the *t*‐value of the correlation,R represents the correlation of the source pair, and K represents the number of data points connected. In our study, the $T_{P}$ value corresponding to a *p* < .05 was used as the threshold to obtain the FC network.

### 2.7 GT analysis

A graph in GT analysis consists of a number of nodes and lines connecting nodes. Nodes represent things, and lines connecting two points represent the relationship between these two things (Maldjian et al., 2014; Rubinov & Sporns, 2010). In this study, we used GT analysis to quantitatively compare the neural networks of the BECTS group versus the control group. The magnetic source in the network was the node, and the functional connection was the line connecting the nodes. We then calculated the average of the four parameters of the corresponding neural network at each frequency band, namely the clustering coefficient (C), degree (D), path length (L), and connectivity strength (S), and then, the differences between the BECTS group and the control group were compared using the same threshold (Kruschwitz et al., 2015).

#### 2.7.1 Strength

In a weighted network G, the connection strength ($S_{i}$) of node "*i*" is defined as the sum of the weight values of the edges directly connected to it (Reijneveld et al., 2007):

(3) $$S_{i}=\frac{1}{N}\sum_{i=1}^{N}w_{\mathrm{ij}}$$

and the strength of the network ($S_{A}$) is the average of the connection strength of all nodes:

(4) $$S_{A}=\frac{1}{N}\sum_{i=1}^{N}S_{i}$$

#### 2.7.2 Degree

The degree of a node represents the number of nodes directly connected to it and can be used to evaluate the importance of a node in the network. For a weighted network G containing N nodes and K edges, the network degree ($D_{A}$) represents the average of the degrees of all nodes in the network:

(5) $$D_{A}=\frac{1}{N}\sum_{i=1}^{N}d_{i}$$

where, $D_{A}$ is an important indicator of network development and compliance (Rubinov & Sporns, 2010; Wang et al., 2016).

#### 2.7.3 Path length

The path length between two nodes "*i*" and "*j*" is the sum of the number of edges the path connecting these two points passes through and the length of each edge is equal to the inverse of the weight value of the edge, namely 1/$w_{\mathrm{ij}}$. The characteristic path length between two nodes "*i*" and "*j*", $L_{\mathrm{ij}}$, is the number of edges of the shortest path connecting two points (Reijneveld et al., 2007). The average value of the characteristic path length of all node pairs in the network is the average path length ($L_{A}$) of the network:

(6) $$L_{A}=\frac{1}{1/\left(N\left(N‐1\right)\right)\sum_{j\neq i}1/L_{\mathrm{ij}}}$$

$L_{A}$ reflects the global characteristics of the network.

#### 2.7.4 Clustering coefficient

The clustering coefficient is an index to reflect the degree of local aggregation of nodes in a graph. Here is the definition: Assume that a node sends k edges, then the maximum number of edges that can exist between the nodes (k) connected by these k edges is k (k − 1)/2, and the score value obtained by dividing the actual number of edges by the maximum number of edges is the clustering coefficient of this node (Reijneveld et al., 2007). For a weighted network G containing N nodes and K edges, the clustering coefficient of node I is determined as the average geometric weight of all nodes:

(7) $$C_{i}=\frac{1}{di\left(d_{i}‐1\right)}\sum_{j,k\in Gj,k\neq i}{\left(w_{\mathrm{ij}}\cdot w_{\mathrm{jk}}\cdot w_{\mathrm{ki}}\right)}^{1/3}$$

where, di represents the number of edges that the node i is directly connected to (the degree of the node i), and w represents the weight of the edges that connect the two nodes. The clustering coefficient ($C_{A}$) of network G is defined as the average of the clustering coefficient of all nodes in the network:

(8) $$C_{A}=\frac{1}{N}\sum_{i=1}^{N}C_{i}$$

$C_{A}$ reflects the local characteristics of the network.

All of the above data analysis was done using the MEG Processor software (https://sites.google.com/site/braincloudx/).

### 2.8 Statistical analysis

The differences in neural FC network patterns between BECTS patients and healthy controls were analyzed using the Fisher exact probability method and the evaluation of data distribution using Kolmogorov–Smirnov test. In addition, the independent sample *t* test was used to compare the differences in intelligence test scores and neural network parameters between BECTS patients and control groups. A Pearson correlation was also used to calculate the correlation between intelligence test scores and abnormal neural network parameters in the BECTS group. Given that strength, path length, and clustering coefficients present a non‐normal distribution, we used the Mann–Whitney U test for comparison and used the Spearman correlation analysis to calculate the correlation mentioned above. Moreover, the Spearman correlation was used to analyze the correlation between the clinical characteristics (age, history of epileptic seizure, and number of seizures) of children with BECTS and the parameters of neural network in the MEG test. Each comparison received a significance level of 0.05 (two‐tailed) and errors were corrected between multiple comparisons using the false discovery rate (FDR) method:

(9) $$pFDR=p_{s}*i/N$$

where $p_{s}$ equals 0.05; N represents the number of tests; i refers to the sorting exponent ($p_{i}$) of the calculated p value ($p_{i};i$ of the minimum p value is 1; the maximum p value i is N; and if there are several of the same p values, then i is the average of these sorting exponentials. The statistical analysis was generated using the SPSS24.0 software package (SPSS Inc., Chicago, IL, USA).

## 3 RESULTS

### 3.1 Subjects

The gender ratio of patients in this study was 11:11 (male: female), and the age of onset was 8.14 ± 1.73 years (see Table 1 **)**. The mean age of the control group was 7.94 ± 1.89 years, and the gender ratio of the control group was 10:8 (male: female). There were no statistically significant differences in gender and age between the two groups. The two groups of children were enrolled in school at the time stipulated by China, and there was no long‐term leave or suspension of schooling. All participants were educated in formal state public schools. There was no statistical difference in the years of formal education.

### 3.2 The WISC‐IV Score of the BECTS Group versus the Control Group

**FIGURE 1:**
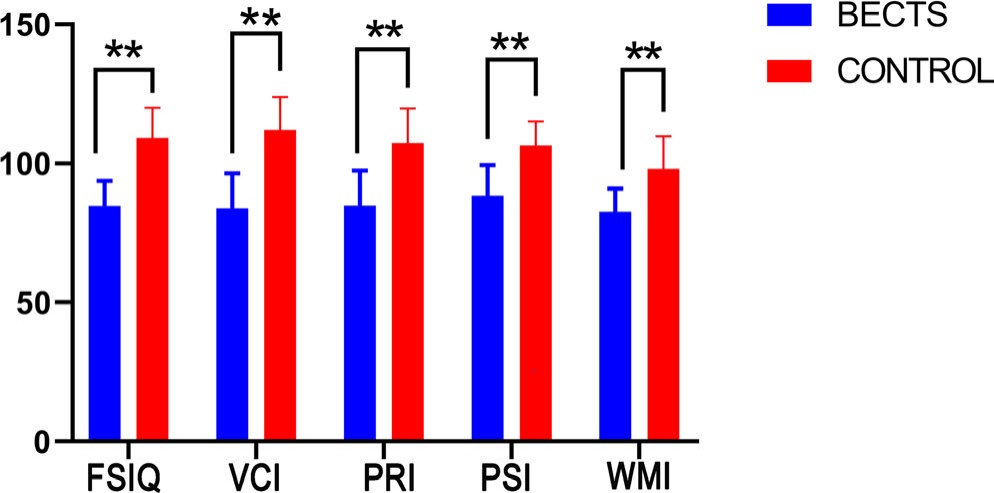
Comparison of WISC‐IV scores between BECTS patients and healthy controls. VCI, verbal comprehension index; PRI, perceptual reasoning index; WMI, working memory index; PSI, processing speed index; FSIQ, full‐scale intelligence quotient. ^∗^ *p* < .05, ^∗∗^ *p* < .01

Children with BECTS presented lower VCI, PRI, WMI, PSI, and FSIQ levels (*p* < .001) than the control group, expressed as $\bar{x}$ ± s (Table 2, Figure 1).

**TABLE 2:** Comparison of WISC‐IV scores between the BECTS group and the healthy group ($\bar{x}\pm S$)

Groups	VCI^**^	PRI^**^	PSI^**^	WMI^**^	FSIQ^**^
BECTS (*n* = 22)	83.86 ± 12.53	84.82 ± 12.63	88.41 ± 11.02	82.55 ± 8.42	84.64 ± 9.07
Control (*n* = 18)	111.89 ± 11.88	107.33 ± 12.53	101.44 ± 8.60	98.11 ± 11.74	109.22 ± 10.87
t	−7.2	−5.6	−4.9	−5.7	−7.8
*p* value	<.001	<.001	<.001	<.001	<.001

Note

WISC‐IV, Wechsler Intelligence Scale for Children, the fourth edition; VCI, Verbal Comprehension Index; PRI, Perceptual Reasoning Index; WMI, Working Memory Index; PSI, Processing Speed Index; FSIQ, Full‐Scale Intelligence Quotient.

^∗^ *p* < .05.

^∗∗^ *p* < .01.

### 3.3 FC networks

**FIGURE 2:**
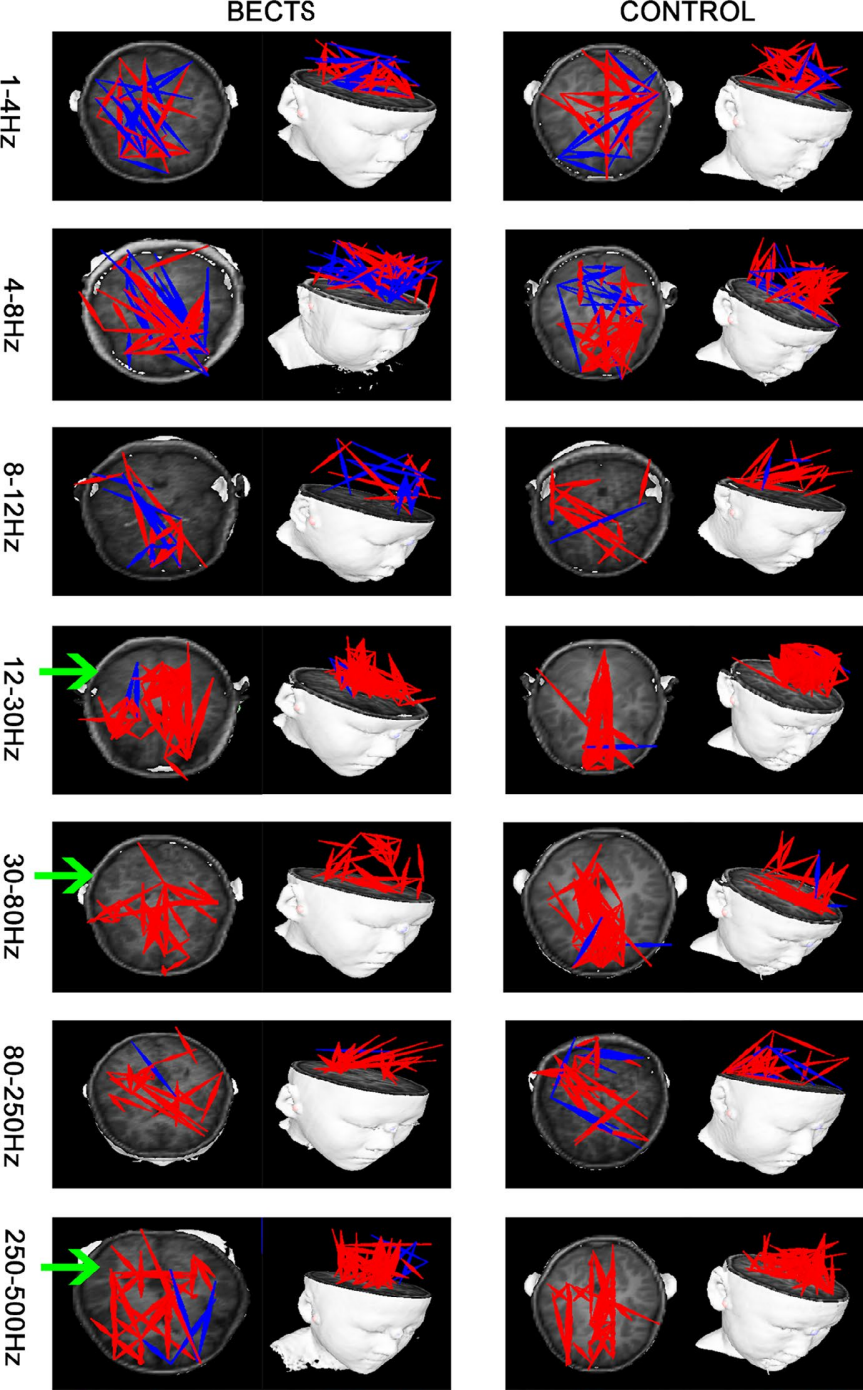
Typical FC patterns for seven frequency bands recorded from BECTS patients versus healthy controls. Red represents excitatory connections and blue represents inhibitory connections. The green arrow indicates a significant difference between the two groups. The above results were corrected using FDR, *p* < .05

Both excitatory and inhibitory connections were detected in our FC analysis. In the 12–30 Hz, 30–80 Hz, and 250–500 Hz frequency bands of the MEG data, FC changed significantly. In the 12–30 Hz frequency band, the FC between the anterior and posterior brain regions of the BECTS patient group appeared weakened, and the FC associated with the frontal lobe was significantly reduced. Furthermore, in the 30–80 Hz frequency band, the FC associated with the frontal lobe was apparently weakened and more dispersed in the BECTS group, when compared to their healthy counterparts. Notably, in the 250–500 HZ frequency band, the FC between the anterior and posterior brain regions of the BECTS group were significantly weakened, and the FC between the brain regions were more dispersed (Figure 2). No significant differences were found in other frequency bands. The above results were corrected using the FDR software and a *p* < .05 was obtained. In our study, we found no correlation between spikes localization and functional network connection change, *p* > .05.

**FIGURE 3:**
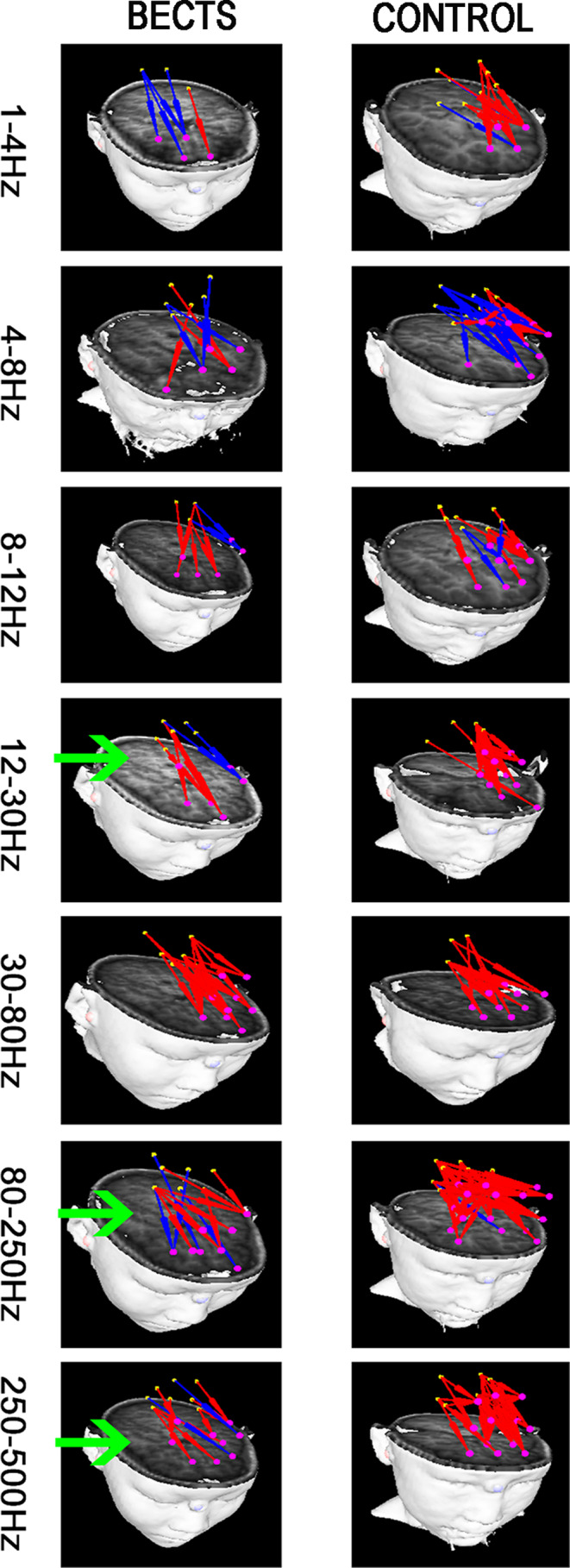
Typical partial FC network pattern of BECTS patients versus healthy controls at 7 frequency bands. The yellow spot is positioned in the posterior cingulate cortex, and the area where the pink spot is located is the frontal cortex. The FC network, which radiated from the posterior cingulate cortex to the frontal cortex, was examined. The green arrow indicates a significant difference between the two groups. The above results were corrected by FDR, *p* < .05

In the analysis of partial functional networks between the frontal lobe and PCC, in the 12–30 Hz, 80–250 Hz, and 250–500 Hz frequency bands, the positive FC network was significantly reduced, and the projection range was narrower than that of the control group (Figure 3). Notably, no significant differences were found in other frequency bands. The above results were corrected using the FDR software, and a *p* < .05 was obtained.

### 3.4 GT analysis

**FIGURE 4:**
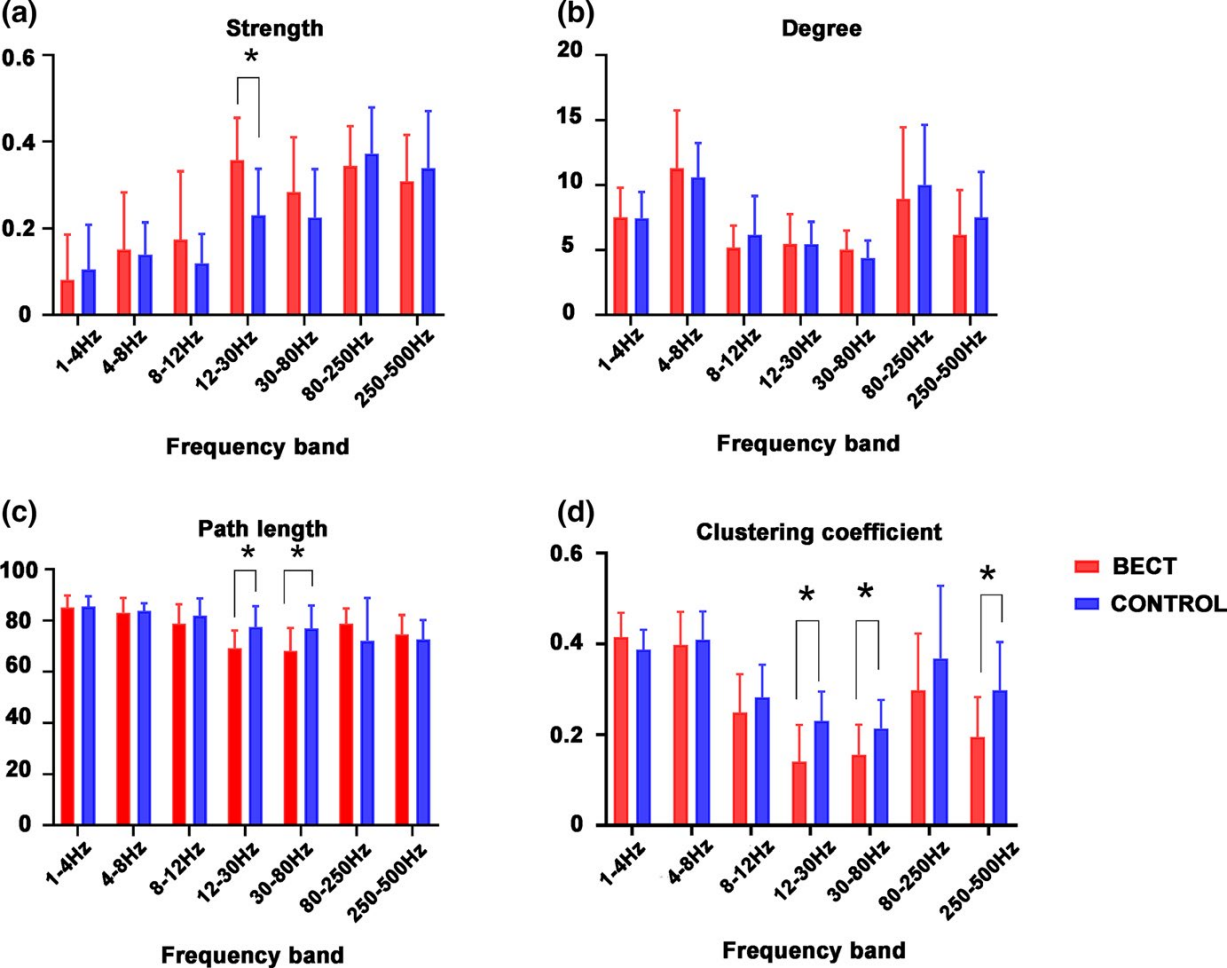
Differences in FC GT parameters (degree strength, path length, and clustering coefficient) between BECTS patients and healthy controls. (a) A comparison of the strength of BECTS patients versus controls. (b) Comparison of the degree of the BECTS patients and controls. (c) A comparison of the path length of BECTS patients versus healthy controls. (d) A comparison of the clustering coefficient of BECTS patients versus healthy controls. The above results were corrected by FDR, *p* < .05

The network GT parameters were obtained by constructing a whole brain functional connection map, including degree, path length, strength and clustering coefficient. For degree analysis, when compared to the control group, no significant differences in each frequency band were observed. Similarly, for strength analysis, when compared with the control group, the BECTS group is significantly higher than the control group in the 12–30 Hz frequency band (*p* < .05), and there were no other significant difference found in other frequency bands. For our path length analysis, when compared to the control group, at 12–30 Hz and 30–80 Hz frequency bands, the path length of the BECTS group was found to be significantly lower than the control group (*p* < .05), and there were no other significant differences found in the other frequency bands. For the clustering coefficient analysis, when compared to the control group, the 12–30 Hz, 30–80, and 250–500 Hz clustering coefficient frequency bands in the BECTS group were significantly lower than those found in the control group *(p* < .05). No significant differences were found in other frequency bands. The above results were corrected using the FDR software (Figure 4), and a *p* < .05 was obtained.

### 3.5 Correlation analysis between WISC‐4 scores and abnormal GT parameters

**FIGURE 5:**
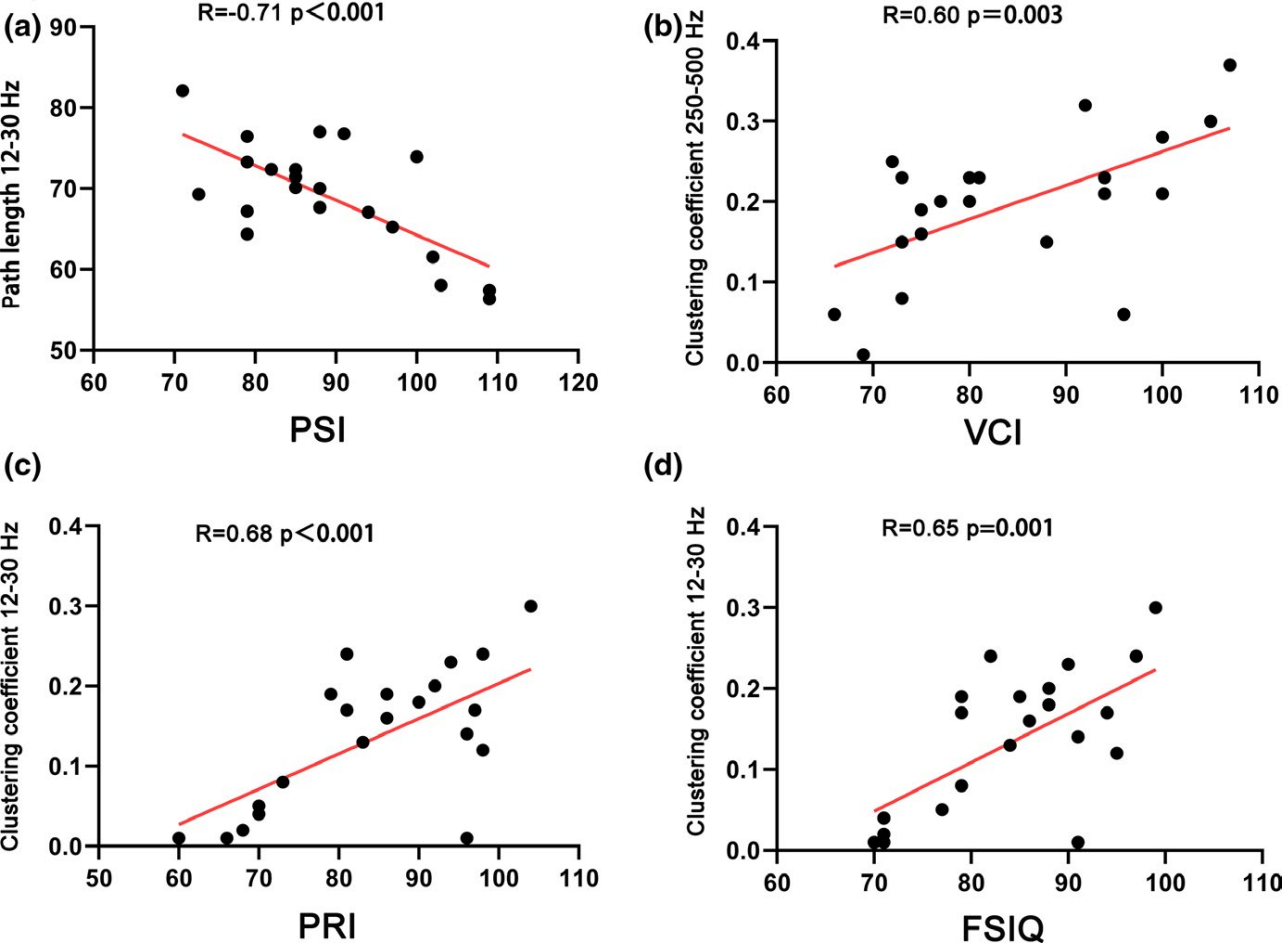
The correlation analysis diagram shows the relationship between FC GT parameters and WICS‐4 scores of BECTS patients in 7 frequency bands. (a) Correlation between PSI and path length at the frequency band of 12–30 Hz (*p* < .001, *R* = ‐.71). (b) Correlation between VCI and the clustering coefficient at the frequency band of 250–500 Hz (*p* = .003. *R* = .60). (c) Correlation between PRI and the clustering coefficient at the frequency band of 12–30 Hz (*p* < .001, *R* = .68). (d) Correlation between the FIQ and clustering coefficient at the frequency band of 12–30 Hz (*p* = .001, *R* = .65) [Correction added on October 19, 2020, after first online publication: The figure 5 legend have been corrected.]

By measuring the Wechsler intelligence test scores of the children evaluated, the FSIQ and four index scores were obtained (VCI, PRI, WMI, and PSI). The correlation analysis was then performed with the abnormal network parameters of the children and children's intelligence test scores. For this, we got the following results: For the FSIQ, in the clustering coefficient value of the BECTS group's MEG 12–30 Hz band, there was a positive correlation (*R* = .65; *p* = .001**); for the VCI, it showed a positive correlation in clustering coefficient values of the 250–500 Hz band MEG in the BECTS group (*R* = .60; *p* = .003**); for the PRI, there was a positive correlation between clustering coefficient values at the 12–30 Hz band in the MEG of the BECTS group (*R* = .68; *p* < .001**); for the PSI, we found that it was negatively correlated with the path value of the 12–30 Hz band in the MEG of the BECTS group (*R* = −.71; *p* < .001**); and finally for the WMI, no significant correlation was seen (Figure 5).

### 3.6 Correlation analysis between clinical data and abnormal GT parameters

We did not find a correlation between age, the course of epilepsy, the number of seizures, and abnormal GT parameters of each frequency band. The results showed no statistical significance.

## 4 DISCUSSION

This study used magnetic brain imaging to study FC networks in the range of low (1–80 Hz) to high (80–500 Hz) frequencies in BECTS patients and measured the WISC‐4 intelligence test scores of these patients, while evaluating the relationship between FC networks and cognitive function. As spike patterns can change and/or resolve over time in BECTS, it is a strength of our study that the patients are relatively newly diagnosed. As far as we know, this is the first time the MEG method is used to explore the relationship between brain FC network and cognitive function in multiple frequency bands that simulate physiological brain magnetism.

### 4.1 The WISC‐IV score

The WISC assessment results showed that the FSIQ, VCI, PRI, and WMI scores of children with BECTS were significantly lower than those on the control group, and the differences were statistically significant (*p* < .01). In our study, the mean FSIQ and some other scores of children with BECTS were lower than average (90–110). Most of the children with BECTS in this study had lower intelligence scores than normal, only a small percentage of children scored in the normal range on intelligence tests, but at the lower end of the normal range. These results indicated that BECTS children have early cognitive impairment, which is roughly the same as previous studies (Vannest et al., 2015; Yan et al., 2017). Some studies have confirmed that the change of brain network is the cause of the onset of epilepsy, and the change of brain network even occurs before the seizure (Garcia‐Ramos et al., 2019). Epilepsy is a connective disease. Brain alterations, epilepsy, and behavioral, cognitive, psychiatric disorders could be epiphenomena, epilepsy‐related variables, brain alterations, and behavioral, cognitive, and psychiatric impairments could be associated with a cause‐effect relationship (Vannest et al., 2015). Notably, seizures and cognitive impairment often appear simultaneously and the results of numerous studies today still do not fully confirm the sequence of epileptic symptoms and cognitive impairment. Some scholars believe that cognitive impairment is a complication of epilepsy and repeated abnormalities in spike waves discharge is the cause of the impairment. Interestingly, some scholars believe that cognitive impairment occurs before seizures and is a precursor to seizures. The sequence in which this takes place has not yet reached a unified conclusion, and it has become a focus of debate (Magara et al., 2019; Vannest et al., 2015; Verrotti et al., 2011). Our results indicate that there is a trend of cognitive impairment in patients with early BECTS. It is speculated that cognitive impairment may be an accompanying symptom or a precursor symptom of BECTS, and its molecular pathophysiology needs to be further studied.

### 4.2 FC networks

In the current literature, more and more studies show that BECTS patients have changes in brain functional connectivity and structural connectivity. These changes may lead to cognitive impairment in BECTS patients, especially in terms of language and executive function (Ciumas et al., 2014; Kagitani‐Shimono et al., 2018; Kim et al., 2014; Luo et al., 2016; Pardoe et al., 2013). In our study, we collected MEG data of BECTS patients in the resting state, and our results show that the FC networks of BECTS patients undergo changes in different frequency bands (Figure 3). Importantly, FC networks have a certain frequency dependence. The specific changes of the brain network in different frequency bands may be caused by epileptic spike discharge. The specific physiological mechanism still needs to be further explored. The relationship between the change of brain network in specific frequency band and the change of cognitive function still needs further research.

In this study, the FC network involving the frontal lobe of BECTS patients changed significantly (*p* < .05), indicating that the brain's DMN in BECTS patients may change during the resting state, a finding which is consistent with previous studies (Luo et al., 2016; Vannest et al., 2013). The frontal lobe is a key area of the brain related to cognitive function, especially executive function (Douw et al., 2011). Here, it shows that the activation of brain regions related to cognitive function are weakened during the resting state of the seizure and that the threshold of the brain area for excitement may be related to the decline of children's cognitive function.

In this study, the FC from the PCC region to the frontal lobe was specifically studied, which also confirmed data from the above‐mentioned study. In the low‐frequency band (12–30 Hz) and the high‐frequency band (80–500 Hz), the FC network of BECTS patients radiating from the PCC to the frontal lobe was weakened (Figure 4). This also shows that BECTS patients may show changes in the DMN in the early stage of the disease. The PCC and frontal lobe are key brain regions of DMN region. The PCC and frontal lobes are key brain regions in the DMN region, and DMN has been considered that associated with cognitive function in previous studies (Chen et al., 2018). This partial change in the FC network may be the reason why BECTS patients have lower intelligence test scores. It has been reported that low‐frequency neural signals may play an important role in large‐scale cortical integration, especially in terms of cognitive function (Sauseng & Klimesch, 2008), and there are also few reports on high‐frequency brain. Our study can be used as a supplement to the high‐frequency neural signal networks in the brain. During an epileptic attack, the presence of spikes in the brain causes changes in the brain's FC patterns, leading to cognitive changes at the onset of BECTS. In the MEG data, we were able to often clearly observe the release of spike waves in children during the interictal at the frequency range of 12–80 Hz, which is consistent with the frequency change of the whole brain FC network mode used in this study. Although the MEG data selected in this study excluded spikes, the potential subsequent effects of spikes discharge on brain function could not be ruled out. Therefore, we speculate that the change in this network pattern may be related to the spikes. In recent years, more and more studies suggest that high‐frequency oscillations (HFOs; >80 Hz) can be used as biomarkers of epileptic foci, and some scholars have proposed that HFOs may be new imaging markers for analyzing brain function and new markers for identifying abnormal brain functions (Haegelen et al., 2013; Jacobs et al., 2012). In our study, we found changes in FC networks at high frequencies (>80 Hz), which validated previously reported data. At the same time, this may be a new imaging marker that prompts cognitive impairment in BECTS patients. Nonetheless, the underlying physiological mechanism needs further research.

### 4.3 GT analysis

Moreover, quantitative analysis of the network using GT shows that BECTS has disrupted the organization of the brain network. This finding indicates that the topological organization in BECTS patients is less optimized in the whole‐brain network. Total strength is the average of the connection strength of all nodes (Rubinov & Sporns, 2010). The increase of this strength can indicate the decrease of interregional connectivity network. Enhanced FC may support the theory that the brain centers of BECTS patients are overexcited during the interictal phase. The decrease in path length and clustering coefficient indicates that children with BECTS have weakened the functional separation and integration of the network topology at the source level. The decrease of path length indicates that the functional integration model between brain regions is complex, the functional association between brain regions is more chaotic, and that the lower the clustering coefficient, the lower the aggregation degree of brain regions. Since these two changes are characterized by a more random topology than the control group, we conclude that the brain network in BECTS patients is developing toward network randomization. These findings could improve our understanding of how recurrent seizures affect the topology of the brain in BECTS patients.

### 4.4 Correlation analysis between WISC‐4 scores and abnormal GT parameters

To clarify the clinical significance of abnormal parameters of MEG data in children with BECTS, we further calculated the correlation between abnormal parameters of MEG and WISC‐4 intelligence test scores in BECTS patients. In this analysis, we found significant correlations between intelligence test scores of children and abnormal parameters of MEG, with the frequency bands of statistical significance mainly concentrated in the low‐frequency range of 12–30 Hz and the high‐frequency range of 250–500 Hz. In the MEG 12–30 Hz frequency band, the FSIQ, PRI, and clustering coefficient are positively correlated, while the PSI and path length are negatively correlated. The clustering coefficient reflects the local features of the brain network, and path length reflects the global features of the brain network (Rubinov & Sporns, 2010). High clustering coefficient and short path length within a certain range can make the brain more efficient in information integration and processing. This is the small‐world property of a healthy brain (Douw et al., 2011). Network changes at the low‐frequency range of 12–30 Hz are closely related to cognitive scores of BECTS patients. Due to FC network changes caused by abnormal spike wave discharge in the brain, the FC network of BECTS patients tends to develop in the direction of randomization, which makes the brain less efficient at processing information. which may be the cause of the cognitive impairment of children with BECTS. This study found that there was a positive proportional relationship between the clustering coefficient and the VCI in the high‐frequency range of 250–500 Hz. Verbal comprehension dysfunction in children with BECTS is a hot topic in the current literature. Perhaps, changes in the FC network in the frequency range of 250–500 Hz may explain this, although it needs further investigation.

Although, we observed several changes in the FC network of BECTS patients, when compared the normal controls, no significant linear correlation was found between these abnormal changes and the clinical data of BECTS patients. Previous studies using resting state functional MRI (fMRI) to study BECTS have reported that changes in functional connectivity are associated with the course of epilepsy (Vannest et al., 2015; Vlooswijk et al., 2010). We speculate that the negative results of this study may be due to limitations of this experiment, such as: (a) The sample size is small, and the differences between individuals are large and (b) The clinical data collected from the children may not be accurate, because many parents do not pay attention to the disease in the early stage, so they cannot provide the exact course of the disease. In addition, the relationship between the clinical data of children and the FC network may not be a simple linear correlation and needs further research.

### 4.5 Limitations

We note that our study still has some limitations. The first limitation is the number of participants. The number of patients we analyzed was limited; therefore, there may be different clinical manifestations and different sites of spike waves in other participants. The relationship between these clinical data and FC network changes of MEG is unclear, and further studies need to be based on a larger cohort of patients. In the meantime, electromyography, EEG, and/or other signals may cause artifacts and errors in our study. Although considerable efforts have been made to minimize these artifacts, further research is needed to determine whether these have been completely eliminated.

## 5 CONCLUSIONS

This study shows that there is a trend of cognitive impairment in patients with early BECTS. In patients with early onset of BECTS, the functional network pattern of resting state has changed, presenting an unbalanced brain topology, and the brain network topology is developing toward randomization. This may be the reason why BECTS patients have a lower cognitive level than healthy children. Changes in the FC network in the MEG 12–80 Hz and the 250–500 Hz frequency band may also provide new imaging biomarker options for assessing the cognitive function of BECTS patients.

## **CONFLICT** OF INTEREST

The authors state that they have no competing interests. They also confirm that they have read the journal's position on ethical publishing and that this report is consistent with those guidelines.

## AUTHORS' CONTRIBUTIONS

Yihan Li analyzed data and wrote this article. Yulei Sun, Tingting Zhang, Qi Shi, Jintao Sun, and Qiqi Chen contributed to data analysis. Zheng Hu and Xiaoshan Wang provided the patients. Jing Xiang provided the MEG software. Xiaoshan Wang was the general responsible person of the project.

### Peer Review

The peer review history for this article is available at https://publons.com/publon/10.1002/brb3.1854.

## Data Availability

The data that support the findings of this study are available on request from the corresponding author. The data are not publicly available due to privacy or ethical restrictions.
